# Successful Treatment of Incomplete Susac Syndrome with Simultaneous Corticosteroids and Plasmapheresis Followed by Rituximab

**DOI:** 10.1155/2021/5591559

**Published:** 2021-08-12

**Authors:** Mick B. Reedy, Yanping Wang, Brad R. Beinlich, William Nicholas Rose

**Affiliations:** ^1^Department of Neurology, University of Wisconsin Hospital, 600 Highland Ave, Madison, WI 53792, USA; ^2^Department of Pathology, University of Wisconsin Hospital, 600 Highland Ave, Madison, WI 53792, USA

## Abstract

We present a case report of a patient with incomplete Susac syndrome. He had cognitive impairment, corpus callosum lesions, and vestibulocochlear dysfunction on brainstem auditory evoked responses. He was treated with methylprednisolone and plasmapheresis, improved, and then, also received rituximab. His improvement has been lasting as of this writing. This case shares our experience with a successful treatment of this rare condition that is incompletely understood and lacks well-established treatment guidelines.

## 1. Introduction

Susac syndrome is a rare microangiopathic disease that is characterized by the clinical triad of central nervous system (CNS) dysfunction, branch retinal artery occlusion (BRAO), and sensorineural hearing loss [1]. Susac syndrome was first described by Susac et al. in 1979 [2]. It typically affects young female patients (20 to 40 years of age) with a female-to-male ratio of 3 : 1 [3]. Clinically, patients often present with headache followed by the development of various other symptoms including vision and/or hearing loss. Because symptoms develop subacutely, fluctuate, and may relapse, the diagnosis of Susac syndrome is often challenging, delayed, or even misdiagnosed.

Susac syndrome is due to an autoimmune-mediated microvascular disease that likely involves cytotoxic CD8+ T-cell-mediated endotheliopathy against the arteries of the brain, retinae, and cochlea [4]. Infarction of these regions plays an important role in the clinical presentation of Susac syndrome. Improvement in symptoms with use of immunosuppressant agents supports the hypothesis that Susac syndrome is immune mediated.

First-line therapy typically consists of high-dose corticosteroids, intravenous immunoglobulin (IVIG), and strong early immunosuppression [5]. Adjunct treatments have been reported for severe or refractory cases, though evidence is lacking. Specifically, the role of plasmapheresis in the treatment of patients with Susac syndrome is not well studied.

We present a case report of a patient with incomplete Susac syndrome who improved after simultaneous high-dose corticosteroids and plasmapheresis and continued to improve after outpatient rituximab induction.

## 2. Case Presentation

The patient is a 65-year-old right-handed male with a past medical history of remote unprovoked deep vein thrombosis (no longer on anticoagulation) and hyperlipidemia who presented to an outside hospital with one week of gait instability and confusion. He had been an avid bicyclist but abruptly lost his ability to maintain his balance on his bicycle. His friends noted significant altered mental status as well, prompting local hospitalization.

On arrival, history was severely limited by encephalopathy, and little collateral was available from family and friends. He lived in a secluded area in the woods. He was very active and spent much of his time outside.

Magnetic resonance imaging (MRI) of the brain with and without contrast showed multifocal, punctate areas of diffusion restriction involving the cerebrum, corpus callosum, and cerebellum involving the cortical gray matter and subcortical white matter. Lumbar puncture (LP) for cerebrospinal fluids (CSF) analysis revealed lymphocytic-predominant pleocytosis (33 cells/*µ*L), absent red blood cells, moderately elevated protein (133 ng/mL), and normal glucose.

He was treated empirically with acyclovir for herpes simplex virus (HSV) encephalitis until CSF HSV polymerase chain reaction (PCR) returned negative. Given positive serum Lyme IgM antibody, he was treated with a full course of ceftriaxone, though his CSF PCR returned negative. CSF Jamestown Canyon Virus returned positive, though this was considered most likely incidental based on his presentation. Routine 30-minute electroencephalogram (EEG) was without epileptiform discharges.

Information about his hospitalization was limited, but he was noted to develop hypersomnolence and abulia. Repeat brain MRI was obtained, showing an increase in multifocal, punctate areas of diffusion restriction in the gray and white matter. Given his clinical deterioration, he was transferred to our center for further investigation.

A broad evaluation for autoimmune, inflammatory, vascular, infectious, neoplastic, and paraneoplastic etiologies was initiated. No evidence of malignancy was present on computed tomography (CT) of his chest, abdomen, and pelvis, nor did testicular ultrasonography have abnormalities to support a paraneoplastic process. Repeat LP was largely unchanged.

Transthoracic and transesophageal echocardiograms were without evidence of patent foramen ovale or vegetations that could lead to cerebral emboli. CT angiography of the head and neck vasculature lacked findings concerning for medium-to-large vessel vasculitis. Diagnostic angiography did not support a clear vasculitic process. Other serum and CSF evaluations were unremarkable. Due to diagnostic uncertainty, brain biopsy was strongly considered, though the family opted not to pursue this.

**Figure 1:**
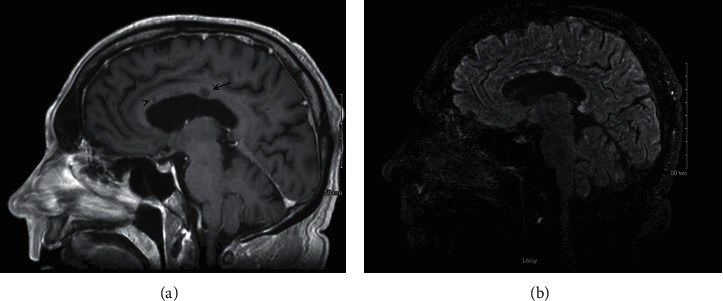
Sagittal views at the level of the corpus callosum during active susac syndrome. (a) T1-weighted. *Note*. The 6-7 mm T1 hypodensity in the body of the corpus callosum was consistent with a “snowball” lesion (demarcated by an arrow), and T1 linear hypodensity sparing the calloseptal surface of the corpus callosum was consistent with a linear “spoke” or “icicle” lesion (demarcated by an arrow head). (b) Sagittal T2/FLAIR-weighted imaging with gadolinium contrast. *Note*. The increased T2/FLAIR signal and contrast enhancement in the “snowball” lesion (demarcated by an arrow) were consistent with active susac syndrome.

Given suspicion for intravascular central nervous system (CNS) lymphoma and primary CNS angiitis, brain MRI was again obtained for consideration of potential brain biopsy sites. Brain MRI again showed new areas of punctate diffusion restriction throughout. At this point, two distinct 4 to 7 mm circumferential T1 hypodensities with associated FLAIR and contrast enhancement were noted in the genu and body of the corpus callosum sparing the callosal undersurface consistent with “snowball” lesions (Figure 1). Furthermore, multiple small linear lesions extending from the roof of the corpus callosum towards central fibers consistent with linear “spoke” (or “icicle”) lesions were noted (Figure 1). These findings were concerning for possible Susac syndrome.

Further investigation of Susac syndrome necessitated retinal and vestibulocochlear evaluation. Retinal involvement was evaluated by optic fluorescein angiography (FA). This study was limited from the patient's profound abulia, though it did not show evidence for BRAO. Audiogram could not be obtained given his abulia; thus, brainstem auditory evoked responses (BAERs) were obtained to evaluate for vestibulocochlear impairment. BAERs showed a primarily peripheral disruption of the auditory pathway on the left and both peripheral and brainstem dysfunction on the right. Whether these findings were chronic in nature could not be delineated. He met the criteria for incomplete Susac syndrome given his constellation of cognitive impairment, corpus callosum lesions on MRI, and central and peripheral vestibulocochlear dysfunction on BAERs.

His symptoms were deemed extremely severe given his severe encephalopathy, cognitive dysfunction, multifocal neurologic deficits, dependence in ADLs, bed-ridden status, and inability to cooperate with FA or audiogram [5]. He was treated with methylprednisolone 1 gm IV for 5 days with continuation of 1 gm weekly after this for a total of 12 weeks.

Simultaneously, a central line was placed for initiation of plasmapheresis (defined here as centrifugal plasmapheresis with a one-volume plasma exchange per procedure using 5% albumin as the replacement fluid). He received a total of 7 plasmapheresis procedures on an approximately Monday, Wednesday, Friday schedule. After the last plasmapheresis, rituximab was initiated as a steroid-sparing agent to decrease antibody production. IVIG was deferred given concomitant plasmapheresis use. Simultaneous immunosuppression with cyclophosphamide, mycophenolate mofetil, and/or tacrolimus was avoided given concern for possible viral infection in conjunction with family preference.

He had gradual improvement in his cognition shortly after treatment initiation. He began inconsistently following basic commands and was discharged to a subacute rehabilitation facility. At his 1-month follow-up, he endorsed overall improvement in cognition, though with frequent fluctuations. Mental status exam showed ongoing disorientation (to month, but not to day of week or year), bradyphrenia, mild global aphasia, amnesia to his hospitalization, and severely impaired recall, though he was able to follow all simple and complex 2-step commands and provide insight into ongoing political news. He continued to need assistance with ambulation, largely secondary to his extensive deconditioning and cognitive dysfunction.

Interestingly, he reported improvement in hearing loss which could not be clinically assessed during his hospitalization. Outpatient audiogram was never obtained due to patient preference. Weekly methylprednisolone infusions were being slowly tapered every 3 months until a 12-month course is completed. Rituximab infusions were scheduled every 6 months for a 2-year course.

At 6-month follow-up, his major deficits included mild cognitive impairment, mild global aphasia, and gait imbalance. While he had improvement in cognitive dysfunction, he continued to require nursing home assistance for management of his activities of daily living (ADLs). He had significant improvement in his gait, but required use of a walker for safe ambulation.

At 9-month follow-up, he endorsed improvement in global aphasia and management of ADLs, though still requiring nursing home care.

**Figure 2:**
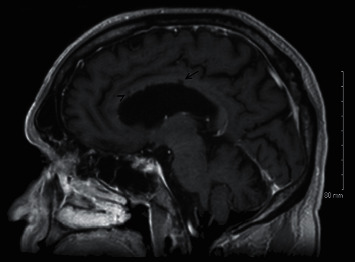
Sagittal T1-weighted slice at the level of the corpus callosum 12 months after treatment initiation. *Note*. The decreased “snowball” lesion in the body of the corpus callosum (demarcated by arrow) and relative persistence of linear “spoke” or “icicle” lesions (demarcated by an arrow head).

Interval MRI brain at 12 months showed reduction or normalization in multifocal areas of punctate diffuse restriction with contrast enhancement including the corpus callosum lesions (Figure 2).

## 3. Discussion

### 3.1. Diagnosis

Susac syndrome is an immune-mediated endotheliopathy that leads to microvascular occlusion and ischemic injury in the brain, retina, and inner ear [6]. The diagnosis of Susac syndrome is based on clinical presentation and neurodiagnostic evaluation. The proposed triad for diagnosing Susac syndrome includes CNS dysfunction, hearing loss, and BRAO. All three criteria are infrequently fulfilled at presentation, with the least frequent being hearing loss, although the triad often becomes fully present after 1 to 2 years [7].

Suspected Susac syndrome refers to female patients without known risk factors for arteriosclerosis or coagulopathy with one symptom of the triad and one of the following risk factors: age 20 to 40 years, within 1 year of pregnancy, and the presence of characteristic corpus callosum lesions or periventricular lesions on MRI [8]. Incomplete Susac syndrome refers to patients with two symptoms of the triad. Complete Susac syndrome refers to patients with all three symptoms.

CNS dysfunction typically consists of acute to subacute encephalopathy with symptoms such as gait abnormality, headache, cognitive impairment, and aphasia [8]. Symptoms range from mild to disabling and may relapse due to fluctuant disease course or with corticosteroid taper. While some studies have found near resolution of cognitive symptoms, there is a wide range of potential cognitive sequelae that may persist indefinitely [9, 10].

MRI of the head with and without gadolinium is frequently used to assess for CNS dysfunction. MRI findings consistent with Susac syndrome classically involve the corpus callosum, showing T2-weighted hyperintensities termed snowball lesions when acute and T1-weighted hyperintensities termed “punched out holes” when chronic; other corpus callosum findings include “icicle” or linear “spoke” lesions [11, 12]. T2-weighted imaging may further demonstrate multifocal hyperintensities in the periventricular, centrum semiovale, cerebellum, and brainstem. MRI is vital in evaluating for other etiologies such as stroke, structural defects, neurodegenerative processes, neoplasm, and metastases. In our patient who had severe encephalopathy and limited history, his pathognomonic corpus callosum lesions were initially dismissed given atypical presentation, numerous multifocal microinfarcts, and concern for infectious etiology. It was only after reexamining these lesions that the investigation for Susac syndrome was pursued.

MRA may be pursued to evaluate for vasculopathy, though being rather insensitive; diagnostic cerebral angiography increases sensitivity for vasculopathy, although it cannot fully rule out small-to-medium vessel arteriopathies [13, 14]. Head and neck vessel imaging is also pertinent to look for sources of embolism [15].

BRAO is evaluated via optic FA [16]. Our patient did not have BRAO, though his examination was limited from cooperativity given his profound encephalopathy. That said, repeat examination on follow-up with appropriate participation failed to reveal BRAO, although BRAO may resolve and become undetectable on FA. Another consideration for evaluation of BRAO in an encephalopathic patient is optical coherence tomography (OCT) [17]. OCT generates high-resolution images of retinal microarchitecture and may be more sensitive for capturing evidence of prior insults during the inactive disease stage.

Hearing loss is typically evaluated with audiograms, though sometimes with caloric testing and vestibular evoked myogenic response (VEMR) [18]. Audiogram was infeasible, and VEMR was not available to us for diagnosis, so we opted to evaluate with BAER. While outpatient audiogram was never obtained, limiting our ability to ascribe his hearing loss to Susac syndrome, his subjective improvement argues in favor of it being a consequence of Susac syndrome rather than secondary to his advanced age.

Other diagnostic modalities are needed to rule out other potential etiologies given our patient's incomplete Susac syndrome. Lab evaluations should evaluate for metabolic derangements, vitamin deficiencies, autoimmune conditions, infections, heavy-metal poisoning, and paraneoplastic syndromes [19]. Susac syndrome is associated with antiendothelial cell antibody (AECA) positivity, though in one study, only about 30% of those with definite Susac syndrome were positive for serum AECA [20]. High-titer IgG1 and IgM AECA in some Susac syndrome patients suggest that humoral autoimmunity may be responsible. That said, AECA seronegativity is common, also suggesting that Susac syndrome may be etiologically heterogeneous and/or AECA is a potential secondary phenomenon following endothelial damage that occurs in a subset of patients. AECA was not tested in our patient.

Routine EEG is important for ruling out seizures, as these can result in transient diffusion restriction in the corpus callosum and encephalopathy. LP with CSF analysis is necessary to rule out infection, vasculitides, paraneoplastic syndromes, and other autoimmune conditions such as neurosarcoidosis, neuro-Behcet syndrome, or Bickerstaff encephalitis [21, 22].

CT imaging may be utilized to search for underlying neoplasm if paraneoplastic syndrome is considered [23]. Echocardiography may be considered to evaluate for cardioembolic sources such as infective or nonbacterial thrombotic endocarditis or left ventricular thrombus [24]. Brain biopsy may be considered if diagnostic uncertainty remains and if there is a reasonable target, but results are typically nonspecific [25].

While our patient was atypical in that he was an elderly male, he met criteria for incomplete Susac syndrome by meeting two of the three criteria in addition to having classic corpus callosum lesions on MRI. Clinical symptoms present to support CNS dysfunction include severe encephalopathy, abulia, and vertigo and were extremely severe. His BAERs supported auditory dysfunction. No BRAO was present on retinal FA. Other candidate pathologies were ruled out with imaging, neurodiagnostics, and lab evaluations. Our case report emphasizes the importance of thorough investigation in undifferentiated cases and shows how pathognomonic findings may be missed in atypical presentations.

### 3.2. Treatment

Susac syndrome is a rare disease without consensus treatment guidelines. The empiric therapy varies depending on the predominant clinical presentation and severity of disease [5]. First-line treatment consists of high-dose corticosteroids, IVIG, and early immunosuppression. Pending severity of disease, response to treatment, and relapse rate, additional immunosuppressants and prolonged courses of corticosteroids and/or IVIG can be pursued. In addition to conventional immunosuppression, alternative treatments including plasmapheresis and stem cell transplant have been reported for severe or refractory cases. While plasmapheresis has been reported for a very small number of patients with refractory Susac syndrome, the role of plasmapheresis in the treatment of patients with Susac syndrome has not been well studied [26].

Given our patient's extremely severe presentation of Susac syndrome, he was started on high-dose methylprednisolone and plasmapheresis with rituximab induction shortly after. Plasmapheresis is a therapeutic procedure that separates blood components and removes offending antibodies [27]. Due to sparse data and the rarity of Susac syndrome, it has not been listed in the American Society for Apheresis guidelines documents [28]. Due to his extremely severe Susac syndrome, plasmapheresis was used as adjunct therapy at the onset of treatment. The relatively low side effect profile also makes plasmapheresis an attractive option, especially considering the high morbidity of this condition.

In one report, 8 of 9 Susac patients improved or stabilized after 5 treatments of plasmapheresis [10]. Because 5 vs. 7 treatments on a roughly Monday, Wednesday, Friday schedule result in nearly identical magnitudes of antibody removal, a schedule of 5 one-volume exchanges is often used for most IgG-mediated illnesses [29]. This common schedule is neither authoritative nor universal [30, 31]. For example, 1-2 additional treatments are sometimes used for autoimmune conditions such as ANCA-associated vasculitis and Goodpasture's syndrome, so it is plausible that these extra procedures helped [28]. In short, given the stakes, acuity, relative safety of the procedure, and the absence of strong evidence, we erred on the side of being relatively more aggressive despite the likelihood of minimal theoretical benefit from procedures 6 and 7.

Our case report is limited in that we cannot infer any clear benefit of plasmapheresis in treating Susac syndrome given the relatively short time period between corticosteroids, plasmapheresis, and rituximab initiation. It may be that steroid initiation coupled with rituximab was sufficient for disease stabilization and recovery. However, the rationale for plasmapheresis is sound due to the theoretical presence of an offending antibody, even if not tested in our patient [32].

## 4. Conclusions

We share our experience with a patient with incomplete Susac syndrome who was treated successfully with simultaneous corticosteroids induction and plasmapheresis followed by rituximab infusions.

## Data Availability

Primary source data are available upon request.

## Abbreviations

CNS: Central nervous system
BRAO: Branch retinal artery occlusion
IVIG: Intravenous immunoglobulin
MRI: Magnetic resonance imaging
LP: Lumbar puncture
CSF: Cerebrospinal fluid
HSV: Herpes simplex virus
PCR: Polymerase chain reaction
EEG: Electroencephalogram
CT: Computed tomography
FA: Fluorescein angiography
BAER: Brainstem auditory evoked responses
OCT: Optical coherence tomography
VEMR: Vestibular evoked myogenic response.
